# Account of a Species of Hydrocephalus, Which Sometimes Takes Place in Cases of Mania

**Published:** 1785

**Authors:** 


### VIII. *Account of a species of Hydrocephalus, which sometimes takes place in Cases of Mania.* By Samuel Foart Simmons, M. D. F. R. S. *Physician to St. Luke's Hospital.*

THERE is a species of Hydrocephalus, which, though not altogether unnoticed by medical writers, does not seem hitherto to have excited a degree of attention proportioned to its importance. The species I allude to, is that in which the water is found not only within the ventricles, but likewise between the pia mater and the surface of the brain.

Such an effusion under the pia mater is indeed mentioned by Morgagni, as having been observed, both by himself and Valsalva, in several cases of what he terms serous apoplexy, and also in two instances of mania; but that it did not attract much of his attention in the latter disease, sufficiently appears from his seeming to consider a præternatural hardness * as the only circumstance that deserved particular notice in the brains of the maniacal patients he had dissected. But the number of such dissec-

* De Sedibus et Caus. Morb. Lib. 1. Epist. 8. Sect 3.

tions, of which we have any account, either in Morgagni's celebrated work, or in the Sepulchretum, is comparatively ſmall *; and as the opportunities my ſituation at St. Luke's hoſpital has afforded me of adding to the obſervations on this ſubject, lead me to ſuſpect that ſuch an effuſion frequently takes place in inſane patients, and to conſider it as a circumſtance likely to prove of conſiderable importance in the pathology of mania, I have thought it right to communicate an account of the following caſes to the public.

### CASE I.

A Shoemaker, aged forty-one years, whoſe inſanity was aſcribed by his friends to hard drinking, was admitted into St. Luke's hoſpital in the year 1781. From the time of his admiſſion, till a few days before his death, though he got but little ſleep, and his pulſe beat from 110 to 120 ſtrokes in a minute, his general health did not ſeem to be materially affected. In his appearance and behaviour he reſembled

* Bonetus has collected only ſix ſuch diſſections; and thoſe related by Morgagni do not exceed that number, if we except the ſingle caſe from Valſalva.

a man a man ſlightly elevated with liquor. He was cheerful and talkative, and ſometimes noiſy, but never miſchievous. At length, however, after having been about ſix months in the hoſpital, he was obſerved to be duller than uſual, and ſomewhat ſtupid. This ſtupidity increaſed during a week or ten days, till one morning he was found, by his keeper, ſpeechleſs, and with one ſide of his body paralytic. At the end of two or three days he recovered his ſpeech, and in a ſlight degree the uſe of his limbs, but ſoon relapſed, and died in about ten days after the firſt attack of palſy.

In the diſſection * of the brain of this patient, nothing preternatural was obſerved on the ſurface of the dura mater, but when this was removed, the pia mater exhibited a remarkable appearance, being of a ſlight milky hue. This was found to be occaſioned by a thickening and opacity of the pia mater, and the effuſion of a limpid fluid between it and

* The diſſection in this and in all the caſes, except the fifth, was made by Mr. Cline. In the fifth caſe, it was performed by Mr. Haighton, aſſiſtant to Mr. Cline in his anatomical lectures. In deſcribing the appearances, I have, in general, confined myſelf to ſuch as ſeemed to be the effect of diſeaſe.

the ſurface of the brain. This ſeparation of the pia mater was ſo conſiderable, that when the upper part of the brain was removed by an horizontal ſection, a ſpace between the pia mater and the brain was every where to be ſeen at the circumvolutions; and in many parts of the right hemiſphere, where this ſpace was moſt remarkable, it was at leaſt a quarter of an inch wide.

The ſubſtance of the cerebrum appeared to be of a natural and healthy conſiſtence. The lateral ventricles contained about four ounces of water.

### CASE II.

AN unmarried woman, aged thirty-eight years, whoſe mother had been inſane, was admitted into the hoſpital in the month of June 1783. About ſix weeks before that time, ſhe had been attacked, while at church, with complaints, which were ſuppoſed to be hyſterical, and the next day the apothecary who was called to her found her reſtleſs and feveriſh, with a quick and ſmall pulſe, and her intellects conſiderably impaired.

At the time of her admiſſion into the hoſpital, her pulſe beat 120 ſtrokes in a minute, and this preternatural quickneſs continued, with more or leſs of increaſe, till her death. Her eyes ſeemed dull, but were in other reſpects of a healthy appearance. She ſpoke but little, and that incoherently; ſlavered inceſſantly; required to be fed by her nurſe; became gradually more and more inattentive to the objects around her, and at length died extremely emaciated in February 1784. i.e. 10 months after y<sup></sup> first attack.

On diſſection, the outer ſurface of the pia mater afforded an appearance ſimilar to that deſcribed in the firſt caſe, though in a ſlighter degree, the quantity of water between it and the brain being leſs. The lateral ventricles did not ſeem to contain a greater quantity of fluid than is uſually to be ſeen in the healthieſt ſubjects; but their capacity ſeemed to be preternaturally enlarged, as were likewiſe the communication between the lateral ventricles, and the iter ad infundibulum, as if they had formerly been diſtended.

### C A S E III.

A Taylor, fifty years old, having been unfortunate in his affairs, became intemperate, and for ſeveral months drank to exceſs of ſpirituous liquors, till at length he became ſuddenly maniacal.

He was brought to the hoſpital in December 1782, after having been inſane about four months. He was a tall, ſtout man, apparently in good general health, and his pulſe beat only 68 ſtrokes in a minute. The pupils of his eyes were in a natural ſtate. He was talkative and incoherent, and got but little ſleep. In the courſe of four or five months he recovered, and was diſcharged from the hoſpital cured in June 1783. He remained well till the month of November following, when he relapſed, and was brought back to the hoſpital about a month after, in a ſtate very different from his former one. His pulſe now beat 120 ſtrokes in a minute; his legs were oedematous; he required to be fed by his keeper, and appeared ſtupid and helpleſs. He remained nearly in this ſtate till his death, which happened in March 1784.

On diſſection, a conſiderable quantity of water was found between the pia mater and the cerebrum, as in the former caſes; and likewiſe, though in a ſmaller proportion, between that membrane and the cerebellum. The ſubſtance of the brain was preternaturally firm, as in the caſes

Hydrocephalus in Maniacs.

cafes defcribed by Morgagni. The lateral ventricles contained about three ounces of water.

### CASE IV.

A labouring man, eighty years old, who was fuddenly attacked with mania in March 1783, was admitted into the hofpital in the month of June following. The account given of him was, that he had been firft attacked with infanity fo long ago as the year 1745, but foon recovered, and had remained well till the prefent attack. The tunica conjunctiva was uncommonly red, but the pupils of his eyes were in a natural ftate. His pulfe beat 84 ftrokes in a minute, and he was in good general health; but he was talkative and noify, required a ftrait waiftcoat, and got but little fleep. He remained in this ftate about three months; after which he became lefs talkative, took but little nourifhment, gradually diminifhed in ftrength and flefh, and died in the month of December, about fix months after his admiffion into the hofpital.

On diffection, the fame opacity and thickening of the pia mater, with a confiderable effufion of water between it and the furface of the brain, brain, were obſerved, as in the former caſes. The lateral ventricles contained about three ounces of water. A ſimilar extravaſation, though in a ſmaller degree, was likewiſe obſervable under the pia mater of the cerebellum. The internal carotid and vertebral arteries were oſſified within the cranium.

### C A S E V.

A Quaker, forty years old, by trade a callico printer, having met with misfortunes in his buſineſs, became at firſt low ſpirited, and, a few weeks after, ſuddenly maniacal. During four or five weeks he was furious, raved inceſſantly, and ſlept but little. After that time, he became leſs noiſy and troubleſome, and when he was admitted into the hoſpital, in February 1785, about ten weeks after the attack of mania, his pulſe beat 120 ſtrokes in a minute, his legs were oedematous, and he was emaciated and ſtupid. From that time he became more feeble and helpleſs till his death, which happened the month following.

On diſſection, the ſame appearances occurred in this as in the preceding caſes, viz. a thickening of the pia mater, and a conſiderable effusion between it and the brain. The lateral ventricles contained only a small quantity of water. The substance of the brain was uncommonly firm, and the septum lucidum seemed to be of more than twice its natural thickness.

### C A S E VI.

A Surgeon's Mate of the Navy, thirty years old, whose brother had been maniacal, was admitted into the hospital in September 1783. He had been insane about three months, but no satisfactory account could be obtained of the manner in which he had been attacked. He was then talkative and incoherent, but recovered in seven months, and remained well till February 1785, when he suddenly relapsed, and being on ship-board, threw himself into the sea. He was re-admitted into the hospital at the beginning of April. His pulse then beat 110 strokes in a minute; he raved incessantly; got no sleep, had a profuse diarrhœa, and was in so emaciated a state, that no hopes were entertained of his recovery.

A little sleep, and short respites from his raving, were obtained by a liberal use of opium, but as soon as the effect of the medicine had subsided, fubfided, he became as noify and furious as ever. Nothing particular was obferved in the ftate of his eyes; nor was his fight affected. He died on the 3d of May.

On diffection, the dura mater appeared in a found ftate; but the pia mater was flightly opaque and thickened, with a fmall quantity of water under it. The lateral ventricles were diftended with about five ounces of water, and from the uniform enlargement of the ventricles, this diftention had probably been flowly effected. The membrane that lines the ventricles, and forms the feptum lucidum, had acquired fo much thicknefs and ftrength, as to be capable of being raifed and ftretched on the handle of a diffecting knife. The pineal gland was enlarged, and of a very foft pulpy fubftance. The brain was of a natural confiftence.

In this, as well as in all the other cafes, the communication between the lateral ventricles, and the third and fourth ventricles, with their openings of communication, were enlarged. The water contained in the ventricles did not coagulate by heat.

The

The foregoing facts will, I flatter myself, be sufficient to excite the attention of physicians, and lead to a farther investigation of the appearances in question; but the number of these facts is as yet too small, and the phenomena of mania involved in too much obscurity to enable us to advance much with certainty on the subject.

These cases, however, form so large a proportion of the maniacal cases it has fallen to my lot to examine by dissection, that I think it probable that a similar effusion will be found in a considerable proportion of those who die of mania.

In all the cases I have related, the pia mater, without the least appearance of inflammation, was more or less thickened and opaque; but at present we can hardly venture to say, whether this thickening of the pia mater deserves to be considered as a cause or as an effect of a morbid excitement of the brain.

That the effusion depends solely on a preternatural hardness of the brain does not seem likely, as such a state of the brain occurred only in two of the six cases I have described. That it may often take place in the mania produced by hard drinking, seems probable, as in two of the preceding cafes the patients were known to have been intemperate in this refpect; and in two other inftances of fuch an effufion related by Morgagni, a fimilar intemperance is mentioned. But from whatever caufes this fpecies of hydrocephalus may originate, the neceffity of endeavouring, as much as poffible, in cafes of mania, to leffen the determination to the brain, and to moderate the preternatural excitement of that organ, feems fufficiently obvious, with a view to prevent an effufion, which, when it has once begun to take place, will, I fear, in general, terminate fatally.

In the hydrocephalus internus of children, where the water is confined folely to the ventricles, we have, in general, fome marks (though oftentimes doubtful ones) characteriftic of the difeafe; but in the fpecies of hydrocephalus which is the fubject of the prefent inquiry, I have not yet been able to difcover any fymptoms fufficiently diftinct from thofe of mania, that can enable us to conjecture, with any degree of certainty, that fuch an effufion has actually taken place.

*Air-Street, Piccadilly,*

June 8, 1785.

SECT.

